# Interprofessional Learning and Improving at the Paediatric Ward: A Participatory Action Research Practising Safety‐II Theory

**DOI:** 10.1111/jep.70061

**Published:** 2025-03-26

**Authors:** Annet van Harten, Margot R. Ernst‐Kruis, Theo J. H. Niessen, Jur J. Koksma, Tineke A. Abma

**Affiliations:** ^1^ Leyden Academy on Vitality and Ageing, Leiden University Medical Centre Leiden The Netherlands; ^2^ Paediatrics Department Meander Medisch Centrum Amersfoort The Netherlands; ^3^ Department of Research Policy Avans University of Applied Sciences Breda The Netherlands; ^4^ Learning Research Group Radboudumc Health Academy, Radboud University Medical Centre Nijmegen The Netherlands

**Keywords:** quality improvement, Safety‐II, participatory action research, stakeholder participation, time pressure, ward‐round, workplace learning

## Abstract

**Rationale:**

In the complex setting of hospitals, professionals often lack time to improve patient safety. Safety‐II theory advocates integrating safety improvement, patient participation, and interprofessional learning and proposes learning frequently from practice variability.

**Aims and Objectives:**

The aim of the research was to understand how interprofessional workplace teams can learn and improve daily from practice variability.

**Method:**

Participatory action research (PAR) was conducted at a Dutch educational hospital paediatric ward to improve situational awareness in bedside ward‐rounds. Methods included 115 semi‐structured interviews and participant observations of the interactions. The action research team consisted of a representation of all stakeholders and the first author, who introduced Safety‐II concepts to reflect on their practice.

**Results:**

The exchange of perspectives between parents, nurses and physicians increased awareness of mutual expectations and experiences prompting individual learning. To foster collective learning at the ward, the research team introduced standards tailored to participants' concerns and stimulated everyday interactions about the ward‐round. This approach facilitated daily mutual perspective taking, expectation alignment, and recognition of practice variability, thereby enhancing unit‐wide learning and improvement. While aiming at increasing shared situational awareness, multiple improvements emerged simultaneously and unexpectedly including time management, professional pride and job satisfaction. However, participants also discovered that lessons learned did not automatically spread to newcomers.

**Conclusion:**

Everyday learning in hospital units can be enhanced through daily interprofessional interactions about expectations and supported by procedural standards. Fostering daily interactions and initiating standards that met participants' concerns required the research team to spend considerable time addressing conflicting priorities. PAR proved to be a valuable and adaptive approach for learning, improving and engaging all stakeholders in a complex setting.

## 1 Introduction

The past decades have witnessed a paradigm shift in healthcare quality and safety [1, 2]. The new paradigm emphasises the vital connection between quality, a culture of continuous learning, and patient involvement [3, 4]. In the new paradigm, often referred to as Safety‐II, hospitals are seen as complex socio‐technical systems and safety is defined as the ability to successfully navigate the stresses and tensions present in modern‐day complex systems [2].

To improve quality and safety, Safety‐II theory advocates to reflect and learn frequently from everyday practice variability that usually results in good care, as opposed to the widespread method of: learning from rare errors, performing root cause analysis and implementing best practices proposed by outsiders [4]. Practice variability refers to the variability in conditions that require variability in work‐as‐done. This differs from practice variety, which is usually associated with non‐compliance and has a negative connotation. However, reflecting every day is not evident in hospital settings where professionals feel that they are already attending too many time‐consuming meetings in addition to their primary duties. This is compounded by staff shortages in these workplaces. Also, it is not common for stakeholders (patients and relatives, nurses and physicians) to reflect together on their shared work processes in hospitals. Safety and core activity will no longer compete for resources if they learn to improve while working on their primary tasks [4]. Then the process of improving will be more sustainable. Also, if an interprofessional team learns to improve while working, it produces its own interventions and adapts them to its circumstances.

Thus, a new safety science paradigm as well as limited reflection time requires hospitals to find ways to learn and improve every day on the job from everyday practice variability, incorporating experiential knowledge of patients. Several studies show the need to integrate patients' experiential knowledge in quality improvement [5, 6, 7, 8]. Advocates of Safety‐II recognise an urgent need to appreciate the frontline efforts directed at improving everyday clinical work. They propose to embed these efforts within the formal organisational learning strategy instead of imposing work as imagined by office staff on the frontline workers [9].

However, several studies have pointed out that complexity is easier to talk about than to act on [10, 11, 12]. Therefore, practical examples of applying complexity theory to improve healthcare are scarce. Practical examples of interprofessional workplace learning with and from patients are equally rare. A recent review on interprofessional collaboration in surgical ward‐rounds concluded that there is a rhetoric on interprofessional collaboration, but that is has not yet become a reality, notwithstanding its relevance for patient safety [13]. Some educational studies have been focused on interprofessional workplace learning [14, 15, 16, 17, 18, 19, 20] and clinical learning environments [21]. However, these studies focus on the clinical learning of individual professionals in the workplace and not so much on the continuous and collective learning of the workplace. Our study aims to fill that gap by looking for tangible, real life, empirical ways a healthcare unit can learn on a collective level to improve quality and safety on an ongoing basis applying safety‐II theory.

This article addresses the question: How can interprofessional workplace teams learn daily from practice variability? In this study, the term ‘interprofessional workplace teams’ refers to both professionals and patients or, in our case, the parents of patients. To answer this explorative question, the researchers needed to start interprofessional daily learning in order to understand its process. In participatory action research (PAR), researchers aim to collaborate with those whose lives or work are at stake to improve their lives which fits our explorative and actionable research question [22, 23].

## 2 Methods

### 2.1 Research Team and Reflexivity

Appendix S1 details the contribution of the co‐researchers to the study. The names of co‐researchers are fictitious except for the authors of the article. M. R. E. K. (second author), paediatrician, and A. V. H. (first author), action researcher and change consultant, were the study's initiators. M. R. E. K. was the project leader and recruited the different co‐researchers: two nurses, a ward manager, a ward physician (resident), a ward supervising paediatrician (educator), a junior consultant from the quality and safety department, and the director of the Foundation for Child and Hospital to guard the perspective of patient and family. All were female except for the paediatrician ward supervisor.

The action researcher is a professional senior change consultant and a PhD candidate. Her educational background is in psychology and business administration. She participated in all stages of the study, mainly by posing questions and holding biweekly online meetings with the project leader. She regularly mailed and telephoned the participating researchers to assess how the research progressed. The action researcher handled most of the data collection (interviews and observations) and facilitated the research meetings. All care providers in the research team participated in the daily ward‐rounds. To ensure the input of experiential knowledge of parents and children, parents were interviewed in their room at the end of every research cycle. All co‐researchers participated in the analysis of the data and the decisions on and preparation of interventions.

Before starting the research, the action researcher and junior researcher spent 1 day at the ward familiarising themselves with the work processes and introducing themselves to participants. The action researcher was familiar with the processes in a hospital ward from her experience as a consultant in another hospital. All participants were informed orally and by an information document about the research and the research team. Telephone numbers were provided in case participants wished for additional information. To the parents, the interviewer was introduced as an expert on quality and safety, who wanted to study how hospital care could be improved.

### 2.2 Design

Within the PAR, researcher A employed also ethnographic data collection methods to describe the work's complexity, contradictions, and conflicts as they appeared to the participants, and their efforts and concerns [24]. The ethnographic methods included observing informal situations and interactions, asking for contextual information about the department, and making field notes of observations and conversations.

The study was multistakeholder and multi‐phased and was organised as pictured in Table 1. Findings from the previous phase were used as input for the succeeding phase. The research project ended after the reflection on action cycle 2. In this reflection, the team decided on the actions for action cycle 3.

**Table 1:** Research design.

Phase	Goals and actions	Data gathering
Introduction Forming the research team and goal setting (June)	Preliminary goal:	Discussion in the research team
	− Improving SA − Learning how to learn daily	
Orientation Describing and analysing the current situation Definitive goal and deciding on actions in the next phase July–September	Reaffirmed goal:	Observations Questionnaires Interviews (at the ward and on the telephone) Small conversations Reading protocols Discussion in the research team
	− Improving SA − Learning how to learn daily	
Action cycle 1 October–December	Actions:	Observations Interviews (at the ward and on the telephone) Small conversations Discussion in the research team
	− Improving SA by poster − Adjusting the time frame − Nurses speak before parents	
Action cycle 2 January–April	Actions:	Observations Interviews (at the ward and by phone) Small conversations Discussion in the research team Member check participants (May)
	− Refining posters and preparation of parents − Adjusted Whiteboard − Use of TRACTUS − Leaving the telephone − Cross‐monitoring and mutual aid	
Action cycle 3	Actions:	
	− Refining actions cycle 2 − Adjusting protocols − Structural periodic interprofessional meetings	

### 2.3 Participant Selection and Setting

The authors chose this paediatric ward of a Dutch educational hospital as the locus of research because it has the characteristics of a social complex system and they were interested in applying the concepts of Safety‐II [25, 26]. The complexity of the paediatric ward arises from its numerous acute, short admissions of patients with varying conditions and diseases. Also, a broad range of experts and parents are involved and there is a fluctuating and unpredictable workload. The patients in this ward were usually too young (under 6 years) to participate.

The chosen ward had 13 one‐patient rooms. The inclusion criteria excluded children from other specialties to enhance research feasibility. Crying babies and anorexia patients were excluded in order not to burden those parents and patients. Some parents had more hospital experience than others.

The research team decided to focus on the daily bedside ward‐round as the opportunity for continuous learning and improving situational awareness (SA) as its improvement goal. The ward‐round is a day‐to‐day work process in which all stakeholders—parents, supervising physicians and residents, nurses and student nurses—participate. The ward‐round has a crucial role in patient safety by creating shared SA. SA involves perceiving and interpreting the situation and anticipating what comes next [27]. It enables teams or units to adapt to unexpected circumstances and act coordinatedly [4]. SA is a core concept in Safety‐II. The word medical visit is specifically used to refer to a single visit to a patient. The ward‐round consists of all medical visits in a day.

### 2.4 Data Collection

Observations and semi‐structured interviews with all participants in the ward‐round were performed every cycle over 2 weeks from Monday to Friday to ensure enough variability in the observed groups. The number of parent interviews per day fluctuated according to the number of patients in the ward.

Research team meetings were audiotaped and transcribed. The transcriptions were not returned for comment. However, the primary action researcher checked her interpretations with the team on an informal basis, which aligns with what Guba and Lincoln call a ‘hermeneutic‐dialectic process’ to prevent bias [28, 29]. The research team learned from the orientation phase that questionnaires caused irritation among the nurses and physicians, and generated low‐quality answers compared to short interview answers. Therefore, they chose semi‐structured interviews instead of questionnaires in the following phases. During the interviews, participants were asked about their expectations, experiences, and wishes for the medical visit. Specific questions were asked about the interventions or changes that had been introduced. The interviews lasted 5–20 min. Parents were interviewed at the ward and via telephone when they returned home. This was to check whether parents rendered different answers once they were home. After two rounds, it appeared he process yielded little additional information. Thus, in the last cycle, parents were only called if they had left the ward before the interviewer visited.

During the research, there were many short informal conversations and interviews about the ward‐round between the participants and the interviewing researchers. In total 82 parents were interviewed during 29 observation days (Table 2). Typically, the interviews with the professionals took place in small groups in the nurse's station or the residents' room. Parents were interviewed in the patients' rooms. Whenever the action researcher engaged with one or more participants, field notes were recorded at the end of the day to reflect on the observations and the role of the action researcher.

**Table 2:** Number of observations and interviews.

Observation days	29
Observed visits	110
Interviewed parents/questionnaires	82
Interviews by telephone	33
Research team meetings	10

### 2.5 Analysis

The research team discussed the observation and interview data after each research cycle. Three authors (A.V.H., J.J.K., T.J.H.N.) analysed the observation and interview data, the transcripts, minutes and field notes. After action cycle 1, data were coded and themes were developed. The codes were compared and grouped, and after some discussion, the authors defined eight relevant themes.

The second coding, after closure of the research, did not lead to additional themes. All five authors participated in further iterative analysis and article writing based on the eight themes. In writing the results section, the authors clustered the eight themes in four section headings (see Appendix S2). The codes, quotations and vignettes were translated from Dutch to English when the article was being drafted.

### 2.6 Authenticity and Trustworthiness

In PAR, the primary criterion is authenticity, whereby the participants recognise and confirm the mutual benefits of the results [22]. This was secured by research team's composition and the collaborative multi‐stakeholder and multi‐phased design of the research process.

The authors enhanced the trustworthiness of the research [30, 31] through four procedures. First, they ensured transferability by providing thick descriptions [32] and quotations evoking ‘vicarious experiences’ [29]. Second, to enhance dependability and confirmability, the authors described the research design and data collection process in detail. Third, reflexivity was sought in all phases of the study by discussing the authors' conceptual lenses, assumptions and the role of the participant‐observer. Fourth, credibility was ensured by the triangulation of methods, data and investigators, and by prolonged engagement and member checking. Finally, the conversations with various stakeholders leveraged multiple perspectives and served the ‘hermeneutic‐dialectic process’ that prevented one‐sidedness and bias of the results [28, 29].

### 2.7 Ethical Issues/Statement

The Institutional Ethics Committee: Medical Research Ethics Committees United (MEC‐U) reviewed the study and determined the Medical Research Involving Human Subjects Act did not apply to this project: Niet‐WMO advies MEC‐U verklaring W21.026.

In addition to confidentiality and informed consent, the following ethical principles were considered: participation, mutual respect, reflexivity, representation and power [33].

Parents received a lay version of the final report. All other participants were given the opportunity to engage in a discussion on the final results in a work meeting.

## 3 Results

The nursing team comprised 40 female nurses of various ages, including student nurses, many of whom had worked in the ward for several years. Two male paediatricians (one more senior than the other) regularly supervised the ward, while eight paediatricians (a mix of men and women of various ages) supervised the ward on a rotational basis. Two residents served as the ward physicians for 3 months, and one intern for 1 week. The ward rounds were conducted 5 days a week at the bedside, involving the participants mentioned above, and parents and patients.

Participants refer to ‘the poster’ and TRACTUS in vignettes and quotations. The poster refers to a coloured laminated A4 material developed by the research team with pictograms and text to remind everybody of the timing, sequential steps and the topics for the medical visit and to encourage parents to write their questions on the whiteboard in the patient room to which the poster was attached. TRACTUS is a mnemonic introduced to clarify what kind of information the physicians wanted to hear from the nurses and in which order to stimulate completeness of information exchange.

### 3.1 Discovering Differences in Expectations on a Daily Basis

During the weeks of data collection, the interviewer often mirrored in informal daily conversations what they had seen in the ward‐round or heard in earlier interviews. This way, the participants discovered that others held expectations, experiences and interpretations other than they thought (Vignette 1).

Vignette 1 **An iterative and mutual process of discovering differences in expectations.**

Some parents did not know precisely what the physician needed from them. As a result, they took the nurses as an example. They shared information on oxygen levels, blood pressure, etc. The professionals realised that some parents needed some information before their first visit to understand the goal and procedure of the visit and the role of their specific experiential knowledge in it. Therefore, the nurses gave the parents more information than in the initial phase.

Nurse Anne: ‘I did not show the poster, but I did tell the parents what was on it and that if they have questions, they can write them down. I think that was clear enough’.

In the subsequent step some nurses discovered that for many parents, just hearing information was not enough. Therefore, they started using the poster when engaging with the parents. Also, they ensured that discharge criteria were written on the whiteboard to help parents remember them and convey the information to absent relatives.

Mother Rianne (after instruction with reference to the poster) mentioned: ‘That poster does help, then you dare to bring it in. In another hospital, I had the experience of forgetting questions every time and then thinking afterward ‘hey, damn, I forgot to ask’. It is very good that it says here: write your questions on the whiteboard. I did that, too’.

Hearing the expectations, experiences and interpretations of others enhanced the readiness to accommodate each other. However, the individual insights and adjusted practices did not spread automatically to others on other days.

### 3.2 Periodically Setting Expectations for the Team

Knowing everyone had different expectations and assumptions about how the work would be done, the research team strove to align these expectations, by formulating standard operating procedures (SOPs) and developing visual aids such as the poster and a TRACTUS checklist.

Getting the research team together for a 1.5‐h meeting proved difficult enough, given the different schedules of those involved, the many projects, and the unpredictability of patient care.

Q1 (Ward Physician Sonja): ‘*The meetings often took a long time when you have other tasks. For me, that was distracting during the meeting. Sometimes, I was already thinking about the work that was left, a physical examination that still needs to be done, e.g., you are with a small team and can therefore not transfer much, both for Rene and me that is difficult*’.

However, spreading the shared goals and SOPs among peers (nurses and physicians) was challenging and time‐consuming, especially in the orientation phase, where many colleagues were sceptical.

Q2 (Nurse Gwendolyn during the orientation phase): ‘*There is only one thing that would really help to speed up decisions, that is the supervising paediatrician always attending the ward‐round. We said that very often before, but they will not do that. So, what's the use of this project?’*

Q3 (Paediatrician Gert): ‘*Is the ward‐round our biggest problem? This PAR approach is very vague; is this science?’*

Q4 (Paediatrician Rene): ‘*In retrospect, I really like being involved in this project, it is insightful. But to be honest, I started the project because someone had to do it. It took a lot of time, which I did not spend on my own project’.*

Additionally, just informing peers was already difficult, especially for the nurses. Only few of the colleagues attended the work meetings and emails were often unread.

Q5 (Paediatrician Margot in the last meeting): ‘*Structure is helpful, but it does take a lot of energy to introduce. Last week, someone still said: Poster? Poster? Are we doing something with a poster?’*

Despite these barriers, the research team succeeded. Vignette 2 describes how.

Vignette 2 **Developing shared goals through constructive conflict.**

**Figure 1:**
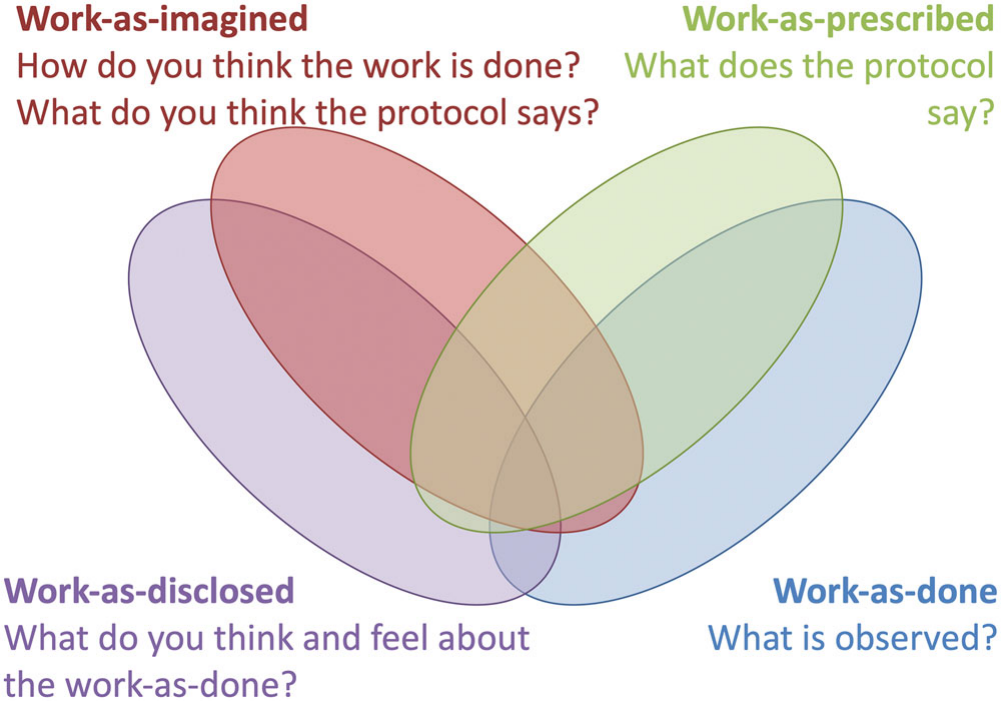
The model used to structure the data of the orientation phase (adapted from Moppet and Shorrock [55]). The model shows that the four domains overlap but also differ.

The data from the orientation phase were presented to the co‐researchers by the first author. For every group (parents, nurses and physicians), the ‘wings’ of the butterfly in Figure 1 was filled with specific examples. This showed the differences in perceptions and priorities between the groups, and the differences between work as done, prescribed, imagined and disclosed. The intention was to understand the differences without judgement.

For the research team, the biggest eye‐opener was seeing differences in their goals or priorities (work‐as‐disclosed) for the ward‐round. The paediatricians took pride in their roles as educators and valued teaching the residents and interns during ward‐rounds. They also appreciated that ward physicians were given the opportunity to conduct ward rounds independently, without the supervision of a paediatrician.

The nurses felt that their expertise was not being used. The parents had already said so much that they had little to add to the issues that were raised. They had to listen to repetitive educational presentations by the physicians and, if the paediatrician was absent, they had to wait whole morning for decisions on discharges.

The parents appreciated that the team took time to listen and explain the process, that the physician took a chair to sit on during the medical visit, and many found the educational presentations interesting.

The research team concluded that all participants shared a common goal: enhancing clarity (situational awareness) regarding essential information about the patients' conditions and the treatment plans. Despite this shared objective, the differences between the groups became more pronounced and more harnessed. There was little exploration, and they disagreed on the conflicting priorities. However, afterward, they tried to accommodate each other. In bilateral conversations among the co‐researchers and a small additional meeting between nurses and physicians they agreed on further arrangements.

**Figure 2:**
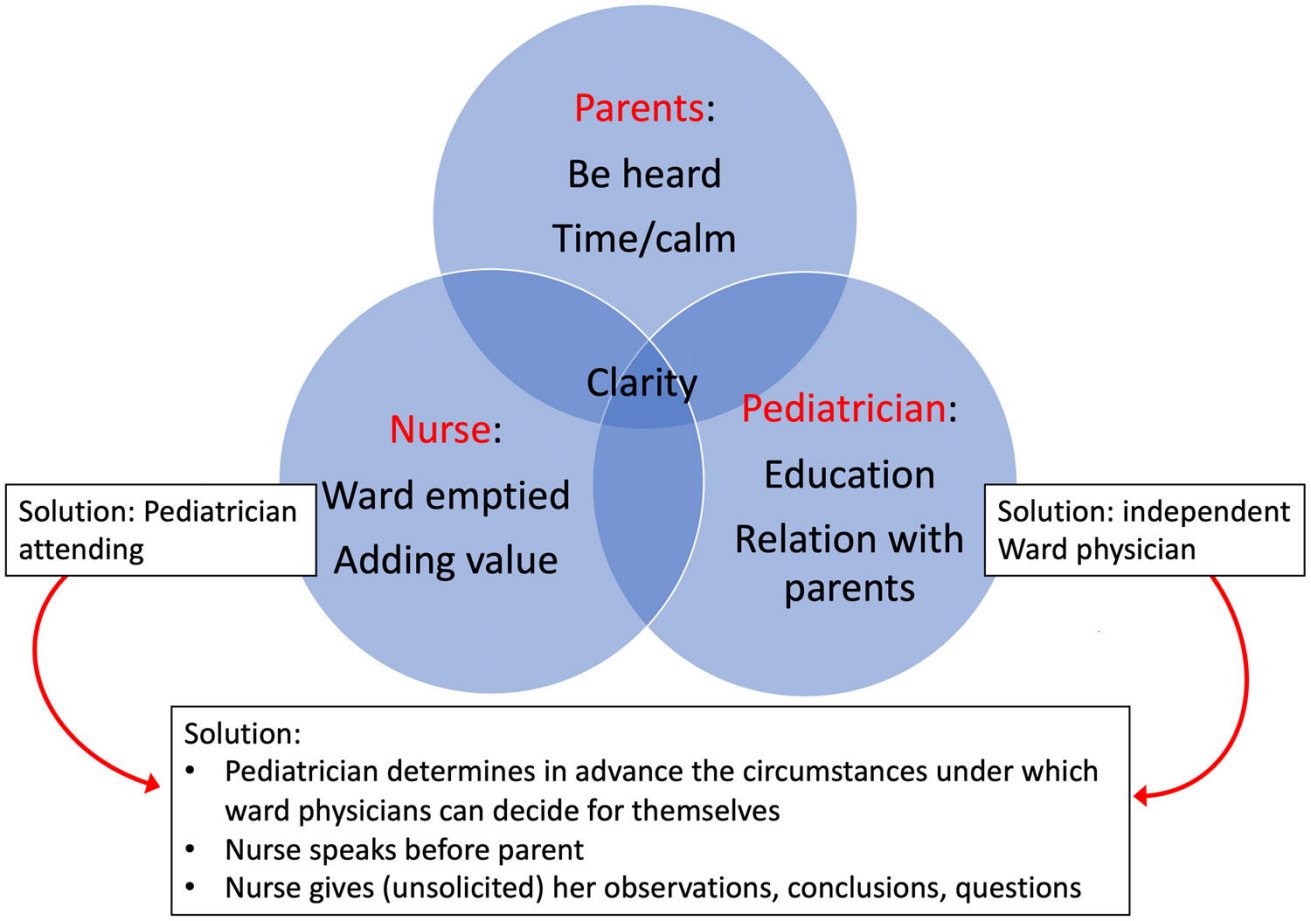
The process of reconciling conflicting priorities. The circles picture the priorities of the participants in the medical visit. Their common priority was clarity. The boxes show how the initial irreconcilable solutions were turned into a solution that was acceptable for all.

Together they found a solution in which patient involvement, education, timely decisions and use of nursing expertise were better balanced (Figure 2).

To the surprise of the physicians, the parents expressed that they were happy to listen to the nurses first and to hear that the nurses had watched over their children well and understood their concerns. The change in order was picked up quickly, even though some nurses found it challenging as it prompted them to prepare more thoroughly first thing in the morning. The change improved the completeness of information shared, clarified expectations, and recognized the nurses' position and expertise.

After each research cycle, the research team followed a consistent pattern: conflicting expectations and concerns were briefly and carefully exchanged during meetings, allowed to settle, and subsequently addressed through additional bilateral dialogues and interventions related to the ward rounds. The topics discussed within the research team reflected the heart felt issues of the broader team (see quotation 6). The proposed solutions were well‐supported, fostering shared goals and expectations among participants and resulting in more improvements than initially anticipated. The research team also gained valuable insights into what they had learned and how they had learned it.

Q6 (Nurse Jennifer): ‘*Because of the discussions after the orientation phase, I especially developed more awareness of the nursing role. I also felt that, […] you can express your own role and be more aware of your own profession that you are trained for. […] Very often we were more caring than nursing’.*

Q7 (Ward physician Sonja): *‘I think it is very important that you do it together and make the plan together. Thus, the process is just as important as the content. Some of the changes are things I have already experienced very positively in other hospitals: the use of the whiteboard, […] the struggle of who do you let speak first. These aspects, I believe, are widely shared. Therefore, it is the process itself that constitutes the learning here. This research created a nice team feeling to work on it’.*

Q8 (Action researcher Annet and nurse Marie): A: *‘Who is going to ask the daily questions about the ward‐round if I'm not there?’* M: *‘We might do that just as well. We can simply ask: What went well today, and what could we do better?’*

Q9 (Ward manager Anneke)*: ‘I didn't know PAR, but now I do not want any other type of research any longer. It creates insight and it includes the involvement of the group. It is investigating and implementing at the same time’.*

The team decided to continue their meetings after the closure of the research every 3 months as Table 1 shows.

### 3.3 Developing an Eye for Variability in Practice by Standards and Cross‐Monitoring

Participants became aware of practice variability after the research team introduced a more standardised way of sharing information and cross‐monitoring by paying attention to the work of other team members instead of just performing one's own task [34, 35].

Q10 (Paediatrician Rene just before the introduction of TRACTUS): ‘*It really is a jumble of information now with all the tracts (organs and functionalities) mixed. Now that I pay attention to it, I notice that even more. It is so nice to be able to improve and change this*’*.*

Initially, the co‐researchers were sceptical about succeeding in cross‐monitoring and offering mutual aid.

Q11 (Ward manager Anneke): *‘It requires communication skills to do so, I don't know if everyone has those skills’.*

In practice, communication skills appeared to be no problem. The barrier was the expectation that others would not appreciate the help offered.

Q12 (Ward physician Jenny): ‘*The nurse was messy and did not have her picture in order and she caused noise about the paracetamol. I am not yet at the stage of giving Gwendolyn feedback on it. If I saw it more often I would. As a ward doctor, we have just been here for one week you know. I also just want to be liked; I'm very honest about that’.*

Q13 (Nurse Gwendolyn): ‘*I didn't feel like I missed things. I did try TRACTUS, but I sometimes get caught up because I react to what is said, and not everything has to be named. The order is illogical for me. Temperature is before paracetamol, but you often relate it to each other. I do have a cheat sheet because I don't have TRACTUS in my head, and I have a note with the vital signs. I keep finding TRACTUS tricky. I am an old hand and used to how things were. But it is good that we all give the same information and that we complement each other. I try to help the ward physician remember, e.g., with patient Janneke, I said to Jenny: Maybe this afternoon a revision at a fixed time?’*

When the interviewer revealed to the interviewees that both ward physicians and nurses were struggling to learn and appreciated help, they started doing so. It created team spirit among participants, including parents, who often reacted with a smile when they observed mutual help.

Q14 (Ward physician Bert): ‘*I felt helped by the nurse, for example when the mother of patient J. expressed nonverbally her concern about the afternoon. The nurse inserted very well by asking the mother ‘are you worried?’. That's good; I might have said that, too. And I think I helped her by noting that the PEWS* [Paediatric Early Warning Signs] *was not zero. Nice that there is a culture where people appreciate the complementation’*.

### 3.4 Spreading Lessons Learned

The research team was surprised to see that in the last action cycle, some improvements—or lessons learned—visible in the first action cycle, such as leaving the telephone behind and writing discharge criteria on the whiteboard, were lost again to some extent in the second action cycle. They had falsely assumed that when learning daily, lessons learned would spread. While individual learning was not impeded, they felt that learning as a team was hindered.

Q15 (Ward physician Sonja): ‘*It is good that the poster is in the onboarding folder, but I think it is ambitious to think that everyone then has an active memory of the poster. As a newcomer, you have so much familiarisation information that you don't have everything ready. You learn by doing and following along. When doing the visit, it should be actively pointed out. […] I was surprised that in the last round it hadn't improved in terms of time management and use of posters. Then you see that it is not so easily transferred between one team and the next’*.

## 4 Discussion

The main research question was, ‘How can interprofessional workplace teams learn daily from practice variability?’ The section below presents the key lessons from the research.

### 4.1 Individual Workplace Learning by Meeting Expectations

One of the main findings is that the exchange of perspectives on ward practices prompted interprofessional learning. This key finding aligns with Oborn's statement that multidisciplinary collaboration is about learning to talk in a new arena [36]. Also, this learning evolves by organising discussions, acknowledging other perspectives and challenging assumptions, a dynamic the researchers also observed in our case.

The implicit premise of this research, in line with publications on Safety‐II [37, 38, 39, 40], was that participants would learn from practice variability. While the introduction of standards improved awareness of practice variability, as quotation 10 illustrates, most of the learning stemmed from interprofessional interactions in which expectations and assumptions were disrupted. Thus, participants did not learn as much from differences in work as done, but rather from differences in work as disclosed.

When expectations and assumptions were disrupted, they were immediately addressed to reestablish alignment. This way, ward physicians and nurses overcame their hesitations to engage in cross‐monitoring and mutual aid. Nurses adjusted their instruction to parents, who in turn accustomed their contribution to the medical visit immediately after the interaction about mutual expectations. These results are consistent with the findings of Snoeren et al. [41].

The authors define workplace learning as ‘the ongoing and relational adapting through the enactment of small and large perturbations in which both agent(s) and environment change and co‐evolve towards enlargement of the space for possible action*’*. This conception of learning builds on the work of Davis and Sumara, who view learning as enacted, happenstance, social and embedded in its context [42, 43, 44, 45]. This offers an additional perspective to the Safety‐II principles that seek to improve complex processes through a structured reflection on practice variability.

In conclusion, the findings suggest that, at least initially, individual participants did not learn from practice variability per se, but from the differing expectations and experiences revealed through communal interactions. These initiated the disruption and immediate adaptation, thereby fostering individual learning.

### 4.2 Workplace Learning as a Unit

A second key finding is that workplace learning as a unit was fostered by the research team initiating interventions that were attuned to participants' needs and concerns and provoked everyday interaction about the ward‐rounds.

In this way, the research team facilitated the transition from dyadic learning, occurring at the level of bilateral or small‐group interactions, to learning at the unit level [46]. This observation also aligns with Snoeren and colleagues's case study in elderly homes [46].

Vignette 2 demonstrates how the non‐judgemental Safety‐II concepts helped the research team bring their different concerns and priorities to the discussion. It also illustrates that the co‐researchers initially did not seem to reflect and learn but harnessed their conflicting priorities. This was found in a study on multidisciplinary learning as well [36, 47]. But by applying a complexity lens to learning [41, 42, 46, 48, 49], the authors perceived in the data an emergent pattern over time in which the professionals in the research team worked on their relations and their conflicting goals at the right time and in the right informal setting. They engaged in constructive conflict [42, 49]. In doing so, they opened up to a process of perspective transformation. This enabled them to develop interventions that effectively balanced patient involvement, education, timely decisions and use of nursing expertise. This way, they addressed their preoccupations and those of their colleagues.

Additionally, because the research team opted for cross‐monitoring, mutual aid and periodic interactive evaluations, as reported in Table 1, they supported all participants in the ward‐round in interacting and changing perspectives. By agreeing to standardise certain practices, they facilitated the perception of practice variability and aligned expectations on how to perform the ward‐round. Consequently, these efforts led to collective learning and ongoing improvements at the unit level.

Notably, literature on interprofessional collaboration often identifies a lack of role clarity, and conflicting priorities and perspectives as barriers to effective collaboration [50]. We redefined these issues as key opportunities to learn and improve as a team (including parents) and develop shared perspectives.

We conclude that the interprofessional learning developed by the co‐researchers through constructive conflicts enabled them to initiate interventions addressing their colleagues' preoccupations, align expectations regarding each other's contributions to the ward round, and encourage daily interactions about their practice.

### 4.3 Time‐Consuming Learning and Time‐Saving Learning

The third and fourth key findings are that interprofessional learning processes proceed non‐linearly and lead to many unexpected but interrelated outcomes, and that the nurses and physicians in the research team experienced the research as time‐consuming.

Findings indicate that, by the end of the research, all co‐researchers felt they had achieved far greater benefits than anticipated. They not only improved shared SA (predefined goal and outcome), but also time management, professional pride for the nurses, work satisfaction and team spirit, a better position for parents and several other practical improvements on the side (see Table 1).

The emergence of unintended results aligns with the nature of change in complex systems. Safety‐II theory recognises that, in complex systems, the components influence one another in a manner similar to an ecosystem—often in unpredictable ways. This is because processes and conditions can resonate and amplify developments, leading to both positive and negative outcomes [51].

The emergence of unintended results and ripple effects was also found in other PAR studies [46]. Abma et al. [22, 23] conclude that it is in line with the nature of change induced by PAR.

Moreover, the co‐researchers acknowledged that they had realised practical improvements and gained insight into learning and improving by fostering daily interactions in the department and by thinking together with all stakeholders in the PAR research team (quotations 6, 7 and 9).

This insight is crucial in the context of ongoing discussions within quality improvement science in healthcare. Dixon‐Woods and Martin [52] advocate for abandoning the pursuit of ‘magic bullet’ interventions and instead emphasising organisational strengthening, as highlighted in high‐reliability studies [53] and resilience research into positive deviance [4].

Despite the PAR yielding more benefits than anticipated, nurses and physicians in the research team found the process time‐consuming. The solutions developed for daily learning had not (yet) resulted in fewer projects or committees, and the ten research team meetings were perceived as an additional commitment. Physicians, in particular, perceived their involvement in the research as time‐demanding (quotations 1 and 4). The difficulty in organising interprofessional meetings within the hospital is a commonly shared finding, stemming from the differing schedules and priorities of participants [50, 54].

Furthermore, the co‐researchers realised that the daily effortless interaction was stimulated by the time‐consuming observations and interviews conducted by the first author, and they were not used to initiating these interactions themselves (quotations 8 and 11). Finally, the co‐researchers noticed that turning new work procedures into a habit took a lot of effort (quotations 5 and 15).

Nevertheless, when harvesting the results at the end of the research, all nurses and physicians in the research team decided they wanted to continue their collaborative safety improvement meetings upon completion of the research (Table 1).

Our initial premise was that integrating learning into everyday practice would ensure that improving safety and core activities would not compete for resources. However, we conclude that while the PAR has been efficient for improving their work, it did not eliminate the perception that improving safety and delivering care competed for resources. The PAR demanded significant time and attention from the research team.

The primary challenge for daily learning is using routine interactions among all stakeholders—parents, nurses and physicians—to discuss not only patient care but also the nature of the work itself. The challenge for periodic interprofessional reflection lies in allocating dedicated time for interprofessional meetings and addressing and navigating conflicting priorities.

## 5 Limitations and Further Research

The researchers performed the study for 1 year in one setting to understand in‐depth the dynamics and possibilities of daily learning. However, a year was too brief to observe whether the learnings the participants formulated would hold after the end of the research. The co‐researchers noticed it was already difficult to bring the residents into the new working methods during the study. A year was also too brief to observe a reduction in projects and committees.

Furthermore, all co‐researchers acknowledged that the action researcher had a decisive role in the daily learning of the team at the workplace and in the research team. However, units usually do not have an action researcher or facilitator at their disposal, which makes it more challenging to translate the findings to other contexts.

Further research will investigate whether the process can be reproduced in other settings, how the role of the external facilitating researcher in the team can be diminished, and how (individual) daily learning in the team can spread across all participants in the unit.

## 6 Practical Implications

PAR might be embedded within the hospitals' formal organisational learning strategy to apply insights from a complexity perspective on quality and safety.

It is a co‐evolving method for learning by action and reflection that fits in a Safety‐II complexity perspective and that flexibly addresses the interrelatedness of safety, patient participation and the frontline efforts to improve everyday clinical work.

## Conflicts of Interest

The authors declare no conflicts of interest.

## Supporting information


***Appendix 1 – Contribution of Co‐researchers***. The roles of all researchers in the research team are described in detail. ***Appendix 2 – Code Tree and Heading Sections***. The codes resulted in eight themes that were translated into four section headings. Table S2 shows how.

## Data Availability

Research data are not shared.
