# Supplementary material for: Interprofessional Learning and Improving at the Paediatric Ward: A Participatory Action Research Practising Safety‐II Theory
Source: J Eval Clin Pract. 2025 Mar 26;31(2):e70061. doi: 10.1111/jep.70061 (PMC11937747; doi:10.1111/jep.70061)
Supplement: Supplementary file 1 — Appendix 1 – Contribution of Co‐researchers . The roles of all researchers in the research team are described in detail. Appendix 2 – Code Tree and Heading Sections . The codes resulted in eight themes that were translated into four section headings. Table S2 shows how. [file JEP-31-0-s001.pdf]

Article title: Interprofessional Learning and Improving at the Paediatric Ward: A Participatory Action Research Practising Safety-II Theory

1. **Annet van Harten** (corresponding author)
Affiliation: Leyden Academy on vitality and aging, Leiden University Medical Centre, Leiden, the Netherlands

Email: annetvanharten@me.com

ORCID: 0000-0002-4213-2373

Phone: +31 6 52000631

Address: Veldstraat 33, 6533 CA Nijmegen, Netherlands

2. Margot R Ernst-Kruis
Affiliation: Pediatrics department, Meander Medisch Centrum, Amersfoort, The Netherlands

Email: MR.Ernst@meandermc.nl

ORCID: 0000-0002-4696-2569

3. Theo J.H. Niessen
Affiliation: Dept. of research policy, Avans University of Applied Sciences, Breda, The Netherlands

Email: theoniessen@gmail.com

ORCID: 0000-0002-8699-0265

4. Jur J. Koksma
Affiliation: Dept. of Research in Learning and Education, Radboud University Medical Centre, Nijmegen, The Netherlands

Email: Jur.Koksma@radboudumc.nl

ORCID: ID 0000-0002-4682-1758

5. Tineke A. Abma
Affiliation: Leyden Academy on vitality and aging, Leiden University Medical Centre, Leiden, the Netherlands

Email: abma@leydenacademy.nl

ORCID: ID 0000-0002-8902-322X

For peer review only

Table S2. Themes and Heading sections

Theme	Results section
1. Informal daily learning	1. Daily discovering differences in expectations
2. Confrontation with variance, other stances	1. Daily discovering differences in expectations
3. The presence of cues to trigger routines	1. Daily discovering differences in expectations
4. Team learning with PDSA	2. Periodically setting expectations for the team
5. Drivers for behavioural change	2. Periodically setting expectations for the team
6. Conditions for perceiving variety.	3. Developing an eye for meaningful differences by standards and cross monitoring (King et al., 2008; Sawyer et al., 2013).
7. Standardisation versus customized action	3. Developing an eye for meaningful differences by standards and cross monitoring.
8. Absence of contagiousness	4. Spreading lessons learned

For peer review only

Article title: Interprofessional Learning and Improving at the Paediatric Ward: A Participatory Action Research Practising Safety-II Theory

1. **Annet van Harten** (corresponding author)
Affiliation: Leyden Academy on vitality and aging, Leiden University Medical Centre, Leiden, the Netherlands

Email: annetvanharten@me.com

ORCID: 0000-0002-4213-2373

Phone: +31 6 52000631

Address: Veldstraat 33, 6533 CA Nijmegen, Netherlands

2. Margot R Ernst-Kruis
Affiliation: Pediatrics department, Meander Medisch Centrum, Amersfoort, The Netherlands

Email: MR.Ernst@meandermc.nl

ORCID: 0000-0002-4696-2569

3. Theo J.H. Niessen
Affiliation: Dept. of research policy, Avans University of Applied Sciences, Breda, The Netherlands

Email: theoniessen@gmail.com

ORCID: 0000-0002-8699-0265

4. Jur J. Koksma
Affiliation: Dept. of Research in Learning and Education, Radboud University Medical Centre, Nijmegen, The Netherlands

Email: Jur.Koksma@radboudumc.nl

ORCID: ID 0000-0002-4682-1758

5. Tineke A. Abma
Affiliation: Leyden Academy on vitality and aging, Leiden University Medical Centre, Leiden, the Netherlands

Email: abma@leydenacademy.nl

ORCID: ID 0000-0002-8902-322X

For peer review only

Table S1. Contribution of Co-researchers

Researchers	Shaping	Generating	Sensemaking	Dissemination
Margot, Paediatrician project leader	Writing grant proposal, developing (initial) design recruiting researchers	Conducting informal conversations and three day observations and interviews. Sharing personal experience	participating in discussions about analysis and actions.	Writing research report for funder and lay language summary. Presenting results on congres, website and magazine.
Annet, Action researcher	Writing first draft grant proposal, developing (initial) design	Conducting observations, interviews and informal conversations, sharing personal experience. Making fieldnotes.	Structuring observation and interview data, providing first analysis Facilitating discussions about analysis and actions	Writing first draft research report and lay summary. Presenting results to researchers from other hospitals.
Iris, Junior quality and safety officer	Making minutes conducting observations and interviews	Conducting observations, and interviews, sharing personal experience	participating in discussions about analysis and actions.	
Rene, Supervising Paediatrician	Thinking along with design, including interview and observation topics	Sharing personal experience	participating in discussions about analysis and actions.	
Hetty & Sonja, Resident paediatrician	Thinking along with design, including interview and observation topics	Sharing personal experience	participating in discussions about analysis and actions.	
Jennifer & Marie, Nurses	Thinking along with design, including interview and observation topics	Sharing personal experience	participating in discussions about analysis and actions.	Disseminating results on congres
Anneke, Ward manager	Thinking along with design, including interview and observation topics	Conducting one day observations and interviews. Sharing personal experience	participating in discussions about analysis and actions.	
Harmke, Director Foundation for Child and Hospital	Thinking along with design, including interview and observation topics Reviewing patient information letters	Sharing experience from similar research projects, garding the patient's perspective	participating in discussions about analysis and actions.	Disseminating results via website and adjusting existing promotional material
Katrijn, Senior officer quality and safety	Writing grant proposal, developing initial design			Reviewing and reacting to final report for funder and to dissemination materials.

For peer review only

The project leader Margot, was responsible for the organisation of the research and she recruited all researchers except for the nurses, who were recruited by the ward manager.

However, after the presentation of the orientation phase two nurses, Jennifer and Marie, brought forward that they felt they should participate in the research team, because they participated on a daily basis in the ward round whereas the recruited nurse seldomly did. Everybody agreed on that, and this coincided with leaving the hospital of the formerly recruited nurse.

Because the residents took up the role of ward physician for only 3 months, Sonja changed places with Hetty. Although in the last phase of the research Sonja was no longer ward physician in this department, she agreed to stay in the research team.

Action researcher Annet was responsible for action research methodology, collecting data, facilitating the research team, and writing.

For peer review only
